# Prevalence, Risk Factors, Harm Perception, and Attitudes Toward E-cigarette Use Among University Students in Qatar: A Cross-Sectional Study

**DOI:** 10.3389/fpubh.2021.682355

**Published:** 2021-08-20

**Authors:** Rana Kurdi, Ghadir Fakhri Al-Jayyousi, Manar Yaseen, Aatefeh Ali, Neama Mosleh, Hanan F. Abdul Rahim

**Affiliations:** Department of Public Health, College of Health Sciences, QU Health, Qatar University, Doha, Qatar

**Keywords:** electronic cigarette, prevalence, knowledge, attitude, practice, Qatar

## Abstract

**Background:** Electronic cigarette (e-cigarette) use is becoming more popular worldwide, especially among youth. Studies report that university students have inadequate knowledge as well as misconceptions about the health risks of e-cigarettes, which may lead to their use even in populations where smoking prevalence is relatively low. At this age, the influence of peers is also significant. Understanding attitudes of university students toward the use of e-cigarettes is important for effective tobacco prevention interventions. In this study, we assess the prevalence of e-cigarette use among students in Qatar's largest national university, as well as their knowledge, attitudes, and perceptions of harm in relation to e-cigarettes.

**Methodology:** We conducted a cross-sectional study among Qatar University students using a self-administered online questionnaire. Descriptive univariate analyses were conducted as well as bivariate analyses to check the association of e-cigarette use with variables of interest. A binary logistic regression analysis was conducted to assess determinants of e-cigarette use among students.

**Results:** One hundred ninety-nine students completed the questionnaire. The prevalence of e-cigarette use among students was 14%, with no significant difference by gender (16.2% in males and 12.8% in females). In bivariate analyses, significantly fewer e-cigarette users believed that e-cigarettes cause disease compared to non-users. 67.9% of e-cigarette users compared to 37.6% of non-users believed that e-cigarettes were less harmful than traditional cigarettes, and 78.6% of users compared to 40.4% of non-users believed that their use could be helpful in preventing smoking traditional cigarettes. Bivariate associations between e-cigarette use and knowledge items were significant (*p* < 0.05) as well as having a smoker among siblings or friends. In the multivariate analysis, only having a friend who was a smoker remained significant after controlling for other variables (OR = 7.3, *p* < 0.001).

**Conclusion:** Our study found that university students have knowledge gaps and misconceptions with regard to the harms associated with e-cigarettes use, especially among users. A comprehensive smoking prevention policy, educational interventions, and quit support are needed to enhance awareness among university students about the health effects associated with e-cigarettes use. Such interventions should also take into account the influence of peers on smoking practices.

## Introduction

E-cigarettes have become widely available since their emergence in 2004, and their use has increased significantly across the world, especially among adolescents and young adults (1, 2). They are often advertised as safer nicotine alternatives or as tools for quitting smoking (3). Moreover, they are produced in a variety of flavors, which particularly enhances their attraction to adolescents (4).

Prevalence of e-cigarette use among university students has been estimated globally through a number of cross-sectional surveys. In Eastern Europe, use of e-cigarettes ranged from 33.4% in Russia and 34.4% in Slovakia to 55.6% in Lithuania (2). In Malaysia, a cross-sectional study using the 2011 version of the Global Adult Tobacco Survey (GATS) tool in six universities found that 74.9% of respondents reported smoking e-cigarettes, and 40.3% reported using both traditional and e-cigarettes (3). In the Middle East, studies among university students in Saudi Arabia reported prevalence levels between 10 and 27.2% (5–9), and a study conducted at five different medical colleges in Pakistan reported that 6.2% of the participants reported using e-cigarettes (10).

Studies to assess the long-term health risks of e-cigarettes are insufficient (2), and there is a need to conduct more cohort studies to confirm their findings (11). Nevertheless, several health risks and side effects have already been documented. E-cigarettes may lead to nicotine addiction similar to conventional cigarettes (6), an increase in heartbeat and blood pressure, and acute inflammatory distress (11). Studies have shown associations of e-cigarette use with asthma (12), lung injury (13), and with a rise in inflammation and oxidative stress, which is a risk factor for atherosclerosis (14). One study, which measured the heart rate variability in e-cigarette users found that e-cigarette use was associated with an increased risk of cardiovascular diseases (14). Smoking e-cigarettes may also cause headaches, cough, chest pain, dizziness (3), irritation of the mouth and throat, nausea, and vomiting (15).

E-cigarette smoking appears to vary by gender. Among current smokers, males are more likely to report smoking and to prefer e-cigarettes over conventional cigarettes compared to females (5, 9). Having peers or family members who smoke was also associated with e-cigarette smoking as shown in a study conducted in Saudi Arabia (5). Another study also conducted in Saudi Arabia reported that peer pressure and curiosity played a major role in e-cigarette use (7). The smoking status of students also influences their e-cigarette use, where e-cigarette users had higher odds of becoming conventional cigarette smokers than non-users (16), possibly because e-cigarette use may be a contributor to nicotine addiction and thus to the initiation of tobacco smoking (16).

Reasons for the popularity of e-cigarette smoking among university students are related to their knowledge and beliefs about the associated risks. A study conducted at Qassim University in Saudi Arabia revealed that university students believed that e- cigarettes were less addictive than conventional cigarettes (5). The knowledge that these students get about e- cigarettes was mostly from social media and advertisement (5). In addition, believing that e-cigarette use help people quit smoking is one of the strongest factors related to usage of this kind of cigarettes among those who did not have enough knowledge about its health risks (7–9).

Qatar is a state in the Arabian Gulf with a population of 2,878,506, among whom 12.1% are aged 15–24 (17). According to the Qatar Global Adult Tobacco Survey (GATS) in 2013, 12.6% of adults living in Qatar were using tobacco, with Non-Qatari males more likely to be smokers (18). Overall, 12.1% were current tobacco smokers, 3.4% were shisha tobacco smokers, 0.7% used smokeless tobacco, and <1% were e-cigarettes users (18).

No studies have been conducted in Qatar about attitudes affecting e-cigarette use among university students. To our knowledge, this study is the first to characterize e-cigarette use among a cross section of students attending the country's largest national university. The objectives of the study are: to assess the prevalence of e-cigarette use among Qatar University students and to examine the factors associated with e-cigarette use, including knowledge of health risk associated with its use, attitudes, perceptions of harm, and the effect of family and friends on e-cigarette uses.

## Materials and Methods

### Study Setting

Qatar University is the oldest and largest national institution of higher education in the State of Qatar. It has a student population of over 20,000 and a faculty body of over 1,000 national and international members (www.qu.edu.qa).

### Study Design, Study Population and Sampling

The study population and methods have been described previously (19) We conducted a self-administered cross-sectional survey on a sample of Qatar University students aged 18–30 years who were actively registered in the spring 2020 semester. Using Cochran's formula, the calculated sample size was 741 students, for a 95% confidence level and a 2.5% margin of error. The assumed proportion of e-cigarette smoking (14%) was based on the findings of a regional cross-sectional study of three universities assessing the prevalence of several tobacco products use among Health Science students in Jeddah-Saudi Arabia (6). We used the lowest prevalence among all tobacco products in that study. The Institutional Research and Analytic Department at Qatar University selected a probability-based sample of students using the proportionate stratified random sampling technique. Students were stratified by nationality (Qatari/non-Qatari) and sex, where 50% of each stratum was selected. In anticipation of the low response rate associated with online self-administered surveys, the questionnaire was sent to 9,807 students, which is half of the number of all the students registered in Qatar University during the specified term. The survey instrument was distributed to the selected students through the Qatar University announcement servers. This study was approved by the Qatar University Institutional Review Board. Data were collected anonymously after completing a consent form.

### Measures

The questionnaire was made available to students in both English and Arabic. Questions were obtained from previous validated questionnaires (3, 5, 7). Questions about tobacco use were adapted from the Global Adult Tobacco Survey (18) and the American Cancer Society's Tobacco-Free Generation Campus Initiative: Cohort 5 Student Survey (2020–2021) (20). We used face validity to assess the accuracy and clarity of questions by piloting the questionnaire with a sample of 20 public health students. All students indicated that the questions were clear, easy to understand, and served the purpose of the study in both languages.

The questionnaire consisted of three parts. The first part assessed socio-demographic characteristics, including gender, age, college, current academic level, marital status, nationality, income, place of living and if they had anyone among family or friends who smoked. It also examined the prevalence and use of different tobacco products. The second part focused on assessing the student's knowledge regarding the influence of e-cigarettes on health, including e-cigarettes as a cause of lung cancer, cardiovascular problems, and cerebral stroke. They also included the following statements: “*e- cigarettes do not contain carcinogenic ingredients,”* “*e- cigarettes are addictive*,” “*e-cigarettes are less harmful to health compared to traditional cigarettes*,” and “*e-cigarettes prevent one from smoking traditional cigarettes*.”

The third part assessed students' attitudes and e-cigarette use. In order to assess respondents' attitudes, we asked respondents about the reasons behind e-cigarette use. The options listed among reasons were: “*I can use them in places where smoking cigarettes isn't allowed;”* “*they might be less harmful to me than smoking cigarettes;*” “*they might be less harmful to people around me than cigarettes;” they might help me quit smoking cigarettes;” “I can use them in places where smoking cigarettes isn't allowed;” “they come in flavors I like;” “they don't smell;” “my friends use them;” “they might help me quit smoking cigarettes;” “I look cool,” and “they are affordable.”* Questions about e-cigarette use included: how many times respondents smoked e-cigarettes during the 30 days before the survey, the most frequently used electronic vapor product used in the 30 days before the survey, and when respondents used electronic vapor products.

A knowledge score was calculated by summing the number of correct answers for all knowledge items. The knowledge score ranged from 0 to 7 depending on the number of correct answers the participant had on the 7 knowledge questions. For each item, a correct answer was given a value of 1 and an “incorrect” or “*don't know*” answer was given a 0 value.

### Statistical Analysis

Data were analyzed using SPSS version 26. Descriptive univariate analysis was run on all variables. Bi-variate analyses (Chi-square/Fisher's Exact test and independent *t*-test/Mann-Whitney test) were conducted to test the associations between e-cigarette use and all demographic variables, between e-cigarette use and knowledge items, and between current smoking status of family or friends and e-cigarette use. All variables found to be significant at the bivariate level were entered into a binary logistic regression model to assess possible determinants of e-cigarette use. A *p* < 0.05 was considered to be significant.

## Results

### Sample Characteristics and Tobacco Use

Out of 741 targeted respondents, only 199 filled the questionnaire. The majority of respondents were female (62.8%), undergraduate students (93.0%), unmarried (83.9%), non-Qatari (58.8%), and living with their families (94.2%). Approximately one third (32.6%) of respondents reported that they had a smoking parent, 33.7% reported having one or more siblings who were smokers, and 48.2% reported having at least one close friend who smokes. Approximately 26% of respondents were themselves current tobacco users (32.4% of males and 21.6% of females), with 15.6% using traditional cigarettes and 14% using e-cigarettes (Table 1).

**Table 1:** Characteristics of respondents.

**Variable**		***N* (%)**
Gender	Male	74 (37.2)
	Female	125 (62.8)
College	Arts and sciences	48 (24.1)
	Health sciences	24 (12.1)
	Medicine	7 (3.5)
	Pharmacy	5 (2.5)
	Dental medicine	1 (.5)
	Sharia and islamic studies	6 (3.0)
	Business and economics	34 (17.1)
	Education	12 (6.0)
	Law	15 (7.5)
	Engineering	47 (23.6)
Current level of education	Undergraduate (B.Sc.)	185 (93.0)
	Masters	12 (6.0)
	PhD	2 (1.0)
Marital status	Single	167 (83.9)
	Married	26 (13.1)
	Other	6 (3.0)
Nationality	Qatari	82 (41.2)
	Non-Qatari Arab nationals	87 (43.7)
	Asian nationals	23 (11.6)
	Other nationality	7 (3.5)
Income (USD)^*^	<2,740	28 (14.5)
	2,740–5,480	52 (26.9)
	5,480–8,219	39 (20.2)
	More than 8,219	74 (38.3)
Place of living	I live with my family	161 (94.2)
	I have my own household	6 (3.5)
	I live in QU student housing	2 (1.2)
	Other	2 (1.2)
Age (years)	Mean ± SD	(23.48 ± 6.86)
	Median ± IQR	(21 ± 4)
Currently using any tobacco product		51 (25.6)
**Type of tobacco product**		31 (15.6)
Traditional cigarettes		
Electronic cigarettes		28 (14)
Chewable tobacco		5 (2.5)
Waterpipe/shisha		36 (18.1)
Others		11 (5.5)
**Current smoking status of family and friends**		
My father is a current smoker		54 (27.1)
My mother is a current smoker		11 (5.5)
One or more of my siblings is a smoker		67 (33.7)
I have at least one close friend who smokes		96 (48.2)
No one in my family is a smoker. None of my close friends is a smoker		59 (29.6)

* *Income levels re-calculated from Qatari Riyals into US Dollars*.

### Knowledge of Health Risks Among All Participants

Although 58.3% of respondents agreed that e-cigarettes may cause lung cancer and 57.8% agreed that e-cigarettes can cause cardiovascular problems, 40.7% did not know that e-cigarettes can cause cerebral stroke, and 50.8% reported that they did not know if e-cigarettes contained carcinogenic ingredients. While 62.8% of respondents reported that e-cigarettes were addictive, 41.9% reported that “*e-cigarettes are less harmful to health compared to traditional cigarettes,”* and 45.7% that “*e-cigarettes can prevent someone from smoking traditional cigarettes.”* The mean (SD) knowledge score was 3.2 (2.15) (Table 2).

**Table 2:** Knowledge about e-cigarettes among all respondents.

**Knowledge**	***N*** **(%)**		
	**Agree**	**Disagree**	**Don't Know**
E-cigarettes can cause lung cancer	116 (58.3)	23 (11.6)	60 (30.2)
E-cigarette can cause cardiovascular problems	115 (57.8)	16 (8.0)	68 (34.2)
E-cigarettes can cause cerebral stroke	95 (47.7)	23 (11.6)	81 (40.7)
E-cigarettes do not contain carcinogenic ingredients	29 (14.6)	69 (34.7)	101 (50.8)
E-cigarettes are addictive	125 (62.8)	28 (14.1)	46 (23.1)
E-cigarettes are less harmful to health compared to traditional cigarettes	83 (41.9)	62 (31.3)	53 (26.8)
E-cigarettes prevents one from smoking traditional cigarettes	91 (45.7)	55 (27.6)	53 (26.6)
Knowledge score (mean ± SD)		3.2 ± 2.15	

### Reasons for E-cigarette Use

Users of e-cigarettes reported that the most common reasons for using them were absence of smell (85.7%), the belief that e-cigarettes were less harmful to the smoker than traditional cigarettes (75%), the belief that e-cigarettes were less harmful than conventional cigarettes to people around them (71.4%), the ability to use e-cigarettes in spaces were traditional cigarettes were not allowed (60.7%), and the availability of e-cigarettes in a variety of flavors (60.7%). Other reasons included affordability (39.3%), the belief that e-cigarettes can help smokers quit (39.3%), use by friends (32.1%), and the image associated with e-cigarettes (32.1%) (Table 3).

**Table 3:** E-cigarette attitudes and practices among e-cigarette users.

**Reasons for using e-cigarette and other vapor products**	***N* (%)**
I use electronic vapor products because they are affordable	11 (39.3%)
I use electronic vapor products because I can use them in places where smoking cigarettes isn't allowed	17 (60.7%)
I use electronic vapor products because they might be less harmful to me than smoking cigarettes	21 (75%)
I use electronic vapor products because they might be less harmful to people around me than cigarettes	20 (71.4%)
I use electronic vapor products because they come in flavors I like	17 (60.7%)
I use electronic vapor products because they might help me quit smoking cigarettes	11 (39.3%)
I use electronic vapor products because they don't smell	24 (85.7%)
I use electronic vapor products because my friends use them	9 (32.1%)
I use electronic vapor products because I look cool	9 (32.1%)
E-cigarette smoking in the 30 days before the survey^*^	
Daily	8 (32.0)
Twice a week	2 (8.0)
Once per week	1 (4.0)
Biweekly	2 (8.0)
Once a month	12 (48.0)
**The electronic vapor product used MOST OFTEN in the 30 days before the survey**	
A device that uses pods	11 (39.3)
A “mod” or device that has a tank	9 (32.1)
A pen-style device	2 (7.1)
A “cig-a-like” device or device that looks like a traditional cigarette	3 (10.7)
The heated tobacco products	3 (10.7)
**Place of using e-cigarettes and other electronic vapor products on campus**	
Indoors	4 (14)
Outdoors	9 (32)
Both indoors and outdoors	3 (11)
I don't use e-cigarettes and electronic vapor products on campus	12 (43)
**Timing of e-cigarette and other electronic vapor product use**	
During university hours	10 (35.7)
In social situations	14 (50)
During stressful situations	15 (53.6)
Other	8 (28.6)
Age of initiation of e-cigarette smoking (mean ± SD)	20.37 ± 8.2

* *There were 3 missing responses for this question*.

### E-cigarette Use

The questions regarding e-cigarettes use indicated that during the 30 days before the survey, 32% of e-cigarette smokers used them on a daily basis. The most popular electronic vapor products used were: a device that uses pods (39.3%) and a “mod” or device that has a tank (32.1%). Almost 10% of the users smoked e-cigarettes and other electronic vapor products indoors on campus, 22% used them outdoors, and 7.3% used them both indoors and outdoors. The majority of users (53.6%) tended to use e-cigarettes and other electronic vapor products during stressful situations and on social occasions (50%). The mean age of starting e-cigarette smoking was 20 years (Table 3).

### Association Between Sociodemographic Variables, Knowledge, Attitude and E-cigarette Use

The median age of e-cigarette smokers was very similar to non-smokers (21 years for both groups). The prevalence of e-cigarette use was 16.2 and 12.8% among males and females, respectively, with no significant difference. Females were more likely to use e-cigarettes, because they “don't smell” (*P* = 0.023).

There was a statistically significant association between e-cigarette use and all knowledge items using a Fisher's exact test (Table 4). Compared to non-users of e-cigarettes, fewer e-cigarette users agreed that e-cigarettes can cause lung cancer (60.2 and 46.4%, respectively), cardiovascular disease (60.2 and 42.9%, respectively), or cerebral stroke (49.1 and 39.3%, respectively). More E-cigarette users believed that e-cigarettes were less harmful than traditional cigarettes compared to non-users (67.9 and 37.6%, respectively), and that their use could be helpful in preventing smoking traditional cigarettes (78.6 and 40.4%, respectively).

**Table 4:** Knowledge and smoking status of family and friends, by e-cigarette use.

	**E-cigarette smokers (** ***N*** **= 28)** ***n*** **(%)**			**Non-e-cigarette smokers (** ***N*** **= 171)** ***n*** **(%)**			*P*-value^*^
	**Agree**	**Disagree**	DK^**^	**Agree**	**Disagree**	DK^**^	
**Knowledge**	13 (46.4)	11(39.3)	4 (14.3)	103 (60.2)	12 (7.0)	56 (32.7)	<0.001
E-cigarettes can cause lung cancer							
E-cigarette can cause cardiovascular problems	12 (42.9)	7 (25.0)	9 (32.1)	103 (60.2)	9 (5.3)	59 (34.5)	0.005
E-cigarettes can cause cerebral stroke	11 (39.3)	10 (35.7)	7 (25.0)	84 (49.1)	13 (7.6)	74 (43.3)	<0.001
E-cigarettes do not contain carcinogenic ingredients	9 (32.1)	6 (21.4)	13(46.4)	20 (11.7)	63 (36.8)	88 (51.5)	0.020
E-cigarettes are addictive	17 (60.7)	8 (28.6)	3 (10.7)	108 (63.2)	20 (11.7)	43 (25.1)	0.038
E-cigarettes are less harmful to health compared to traditional cigarettes	19 (67.9)	7 (25.0)	2 (7.1)	64 (37.6)	55 (32.4)	51 (30.0)	0.006
E-cigarettes prevents one from smoking traditional cigarettes	22 (78.6)	5 (17.9)	1 (3.6)	69 (40.4)	50 (29.2)	52 (30.4)	<0.001
**Knowledge scores out of 7 (mean ± SD)**		2.2 ± 1.7		3.3 ± 2.2			0.041
**Current smoking status of family and friends**							
My father is a current smoker		11 (39.3)			43 (25.1)		0.167
My mother is a current smoker		1 (3.6)			10 (5.8)		1.000
One or more of my siblings is a smoker		15 (53.6)			52 (30.4)		0.029
I have at least one close friend who smokes		24 (85.7)			72 (42.1)		<0.001

* *p-value based on Fisher's Exact test for categorical variables and independent t-test for means*.

** *DK, Don't know*.

E-cigarette smoking was also significantly associated with having a sibling who smokes (*p* = 0.029) who smokes and having at least one close friend who smokes (<0.001). On the other hand, there was no significant association between e-cigarette use and the smoking status of mothers or fathers.

A binary logistic regression was conducted to detect the possible determinants of e-cigarette use. The variables that were found to be significant at the bi-variate analysis level were included in the regression model (knowledge score, having at least one sibling who smoked, and having at least one friend who smoked). Only the friend effect remained significant after controlling for the other variables. The odds of having a friend who smoked were 7.3 among e-cigarette users compared to non-users (*p* < 0.001; Table 5).

**Table 5:** Determinants of e-cigarette use.

	***P*-value**	Adjusted OR (95% CI)^*^
Knowledge score	0.09	0.837 (0.681, 1.029)
Sibling effect^**^	0.074	2.198 (0.926, 5.216)
Friend effect^**^	<0.001	7.298 (2.394, 22.252)

* *Adjusted for the variables in the model*.

** *Reporting having at least one sibling or one friend who smoked*.

## Discussion

To our knowledge, this is the first study that investigated the use, knowledge, and attitudes toward e-cigarette use among university students in Qatar. The study was conducted in the largest national university in the country, and we found the prevalence of e-cigarette use to be 14%. This prevalence is within the range of reported e-cigarette use in the region. It is higher than the prevalence of Qassim University (10%) and that of Alfaisal University in Saudi Arabia (12.2%) (5, 9) and that of the study conducted in Pakistan (6.2%) (9). However, our prevalence was lower than the prevalence found in another three Saudi studies (27.7%), (25%), (21%) (6–8). In the Qatar GATS 2013 survey, only 0.9% of respondents were found to be current e-cigarette users (18). While this difference may be due to the fact that the population in the GATS Qatar survey included adults aged 15–65 years, it is likely that the increase represents a real growth in the popularity of e-cigarettes since 2013, requiring a focus of public health efforts on their rise.

Our findings showed that e-cigarettes are still less popular than conventional cigarettes. However, we also found that 45% of those who smoked traditional cigarettes and 80% of those who used chewable tobacco were concomitantly using e-cigarettes. This finding may indicate that some tobacco users can be trying e-cigarettes at the same time either as an attempt to stop smoking or to satisfy their curiosity. In Pakistan, the tobacco product that was mostly used among university students was conventional tobacco (38.8%) either in the form of cigarettes, shisha, or smokeless tobacco, while only 6.2% of participants confirmed using e- cigarettes (11). However, a study conducted in Saudi Arabia indicated a higher prevalence of e- cigarette use (27.7%) compared to conventional cigarette use (14.1%) among university students, a finding which the authors believe may be due to wide-scale advertisement campaigns targeting young adults (6).

Our study pointed to a significant issue with regard to e-cigarette harm perception. We found that 41.9% of students believed that e-cigarettes were less harmful to health compared to traditional cigarettes, which would encourage students to continue or start using e-cigarettes if they do not receive the needed awareness in regards to health risks associated with this type of tobacco product. These findings align with the findings of one of the Saudi studies, which reported that more than half of the students believed that e-cigarettes were less harmful than conventional cigarettes (7). Moreover, 45.7% of the participants in our study agreed that e-cigarettes prevented one from smoking traditional cigarettes, which implies that this proportion of students may revert to e-cigarette use or advise their peer smokers to revert to this type of smoking. The misperceptions of harm were more pronounced among e-cigarette users compared to non-users, according to our findings, signaling the need for a nuanced dual approach focusing on e-cigarette smokers as well as the non-users.

Participants in our study used e-cigarettes because they do not smell, they might be less harmful than smoking traditional cigarettes to participants and people around them (which again reinforces the spread of misconceptions about e-cigarettes), they can use e- cigarettes in places where smoking cigarettes is not allowed, and they come in many flavors which students like. In comparison to one of the studies conducted in Saudi Arabia, peer pressure, curiosity, and believing that e-cigarette would help them quit smoking were the main reasons for e-cigarette use among university students (7). A study by Qanash et al. indicated that the four main reasons of e-cigarette use among university students were entertainment, peer effect, anxiety and stress relief, and quitting conventional cigarette smoking (6). Another study by Habib et al. conducted also in Saudi Arabia, found that the main reasons for using e-cigarettes were enjoying different flavors, reducing or quitting conventional tobacco smoking, and avoiding the public smoking ban (9).

With reference to e-cigarette use among Qatar university students, half of them used e-cigarettes in social occasions and 32% of them used them on a daily basis. Similarly, a research conducted among health science students in Saudi Arabia found that 79.1% of students were using e-cigarettes occasionally, while only 20.1% of them were using them on a daily basis (6). Moreover, the survey that was conducted among medical students in Pakistan reported that only 1.2% used them daily (11). This indicates that the frequency of daily use is higher in our study compared to other studies in different countries. Our participants used e-cigarettes outdoors (32%), which was similar to a study conducted by Qanash et al. in Saudi Arabia where 21.8% of smokers mentioned that they used them in cafes and restaurants (6). However, more than 17% of the users in our sample used e-cigarettes indoors and outdoors (with 10% used them only in-doors), which might indicate that there is no clear policy on their use on campus. Indeed, this lack of awareness and the need for a clearer and more comprehensive campus tobacco policy has been found in another study of this same population (19).

We found that 32.4% of males and 21.6% of females were current tobacco users. This finding indicates that though males are using tobacco products more than females, it seems that female students are becoming more curious to try some types of tobacco products as the GATS survey conducted in 2013 indicated that only 3.1% of females admitted being smokers (18). Our study also showed that the prevalence of e-cigarette use was higher among males (16.2%) than females (12.8%), but this difference was not statistically significant, which indicates that e-cigarettes use is becoming common among females in Qatar. Interestingly, we found that the main reason encouraging females to use e-cigarettes was that they don't smell. Smoking among females carries a social stigma in Arab culture (7), so it is likely that women prefer e-cigarettes as an apparently more acceptable or less stigmatizing alternative. A study conducted in Malaysia among university students found a relationship between exclusive e-cigarette use and gender where users were mostly males (3). Also, two studies conducted in Saudi Arabia indicated a significant association between e-cigarette use and being a male (7, 9). The authors attributed that to the fact that smoking among females is culturally unacceptable and perceived to be taboo in Saudi Arabia (7).

Our study assessed the association between e-cigarette use and current smoking status of family and friends. The e-cigarette usage was more prevalent among those who had a sibling or at least one close friend who smokes which reinforces the role of peers in initiating smoking. A similar finding was reported in one of the studies conducted in Saudi Arabia by Almutham et al. where students reported that 70.4% of e-cigarette smokers have a family member or a friend who smokes (*P* < 0.001) (5). These findings point to the important influence of peers and family in shaping attitudes and practices related to e-cigarette use, and therefore the need to include them in interventions.

This is the first study to investigate knowledge and attitudes toward e-cigarette use among university students in Qatar. Our study highlighted the fact that the use of e-cigarettes is increasing among young adults in Qatar and that it is associated with lack of knowledge, misconceptions, and peer influence. The findings will enrich the literature about factors influencing the use of various tobacco products, which will in turn support the planning for health education interventions and effective smoking prevention policies on a university campus. However, our study has some significant limitations, including the small sample size and low response rates. Low response rates are not unusual in self-administered online surveys, but they increase the risk of bias and limit generalization. Furthermore, the use of a cross sectional design does not allow clear identification of causal relationships.

Future qualitative research will be helpful in uncovering deep-seated cultural and gender attitudes that shape perception and practice of tobacco use, including e-cigarette use. The findings of such in-depth qualitative work would supplement survey findings and help inform effective interventions.

## Conclusion

E-cigarette use is becoming popular among university students. Inadequate knowledge of the health risks associated with e-cigarettes and misperceptions about e-cigarettes preventing the use of traditional cigarettes require tailored health interventions as well as comprehensive campus tobacco policies to address their harm.

## Data Availability Statement

The datasets presented in this article are not readily available because dataset will be made available upon reasonable request. Requests to access the datasets should be directed to hanan.arahim@qu.edu.qa.

## Ethics Statement

This study was reviewed by Qatar University Institutional Review Board (IRB) and was granted with ethical approval number of QU-IRB 1188-E/19. We obtained informed consent online from participants before proceeding to answer survey questions. Students were assured of the confidentiality of their responses. The patients/participants provided their written informed consent to participate in this study.

## Author Contributions

This research was conducted by public health students as a senior graduation project with the supervision of public health instructors at Qatar University. RK: questionnaire design, statistical analysis, and write-up of the manuscript. HA: grant writer/recipient, conceptualization of the study, questionnaire design, critical review of the manuscript, and response to reviewer comments. GA: conceptualization of the study, questionnaire design, proposal writer and IRB approval recipient of the larger project which this study is part of, sampling design and data collection, and write up of the manuscript. MY, AA, and NM: statistical analysis and write-up of the manuscript supervised by RK. All authors contributed to the article and approved the submitted version.

## Conflict of Interest

The authors declare that the research was conducted in the absence of any commercial or financial relationships that could be construed as a potential conflict of interest.

## Publisher's Note

All claims expressed in this article are solely those of the authors and do not necessarily represent those of their affiliated organizations, or those of the publisher, the editors and the reviewers. Any product that may be evaluated in this article, or claim that may be made by its manufacturer, is not guaranteed or endorsed by the publisher.
